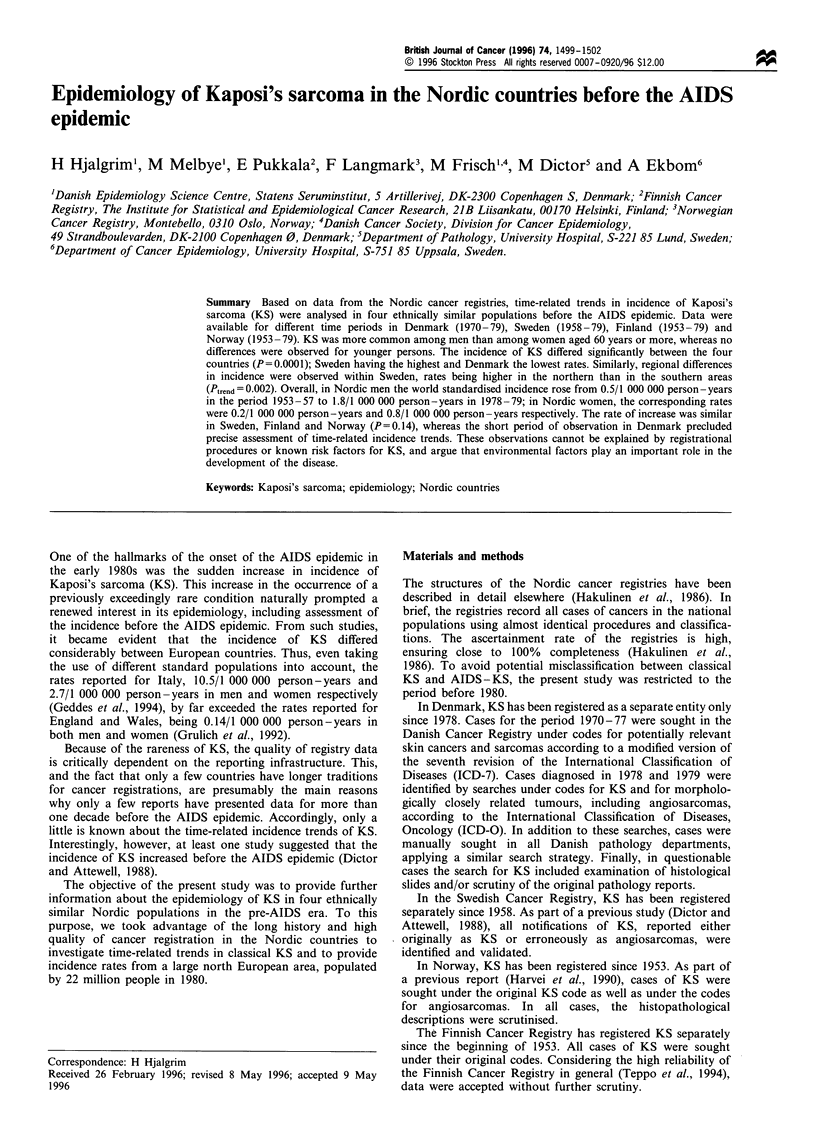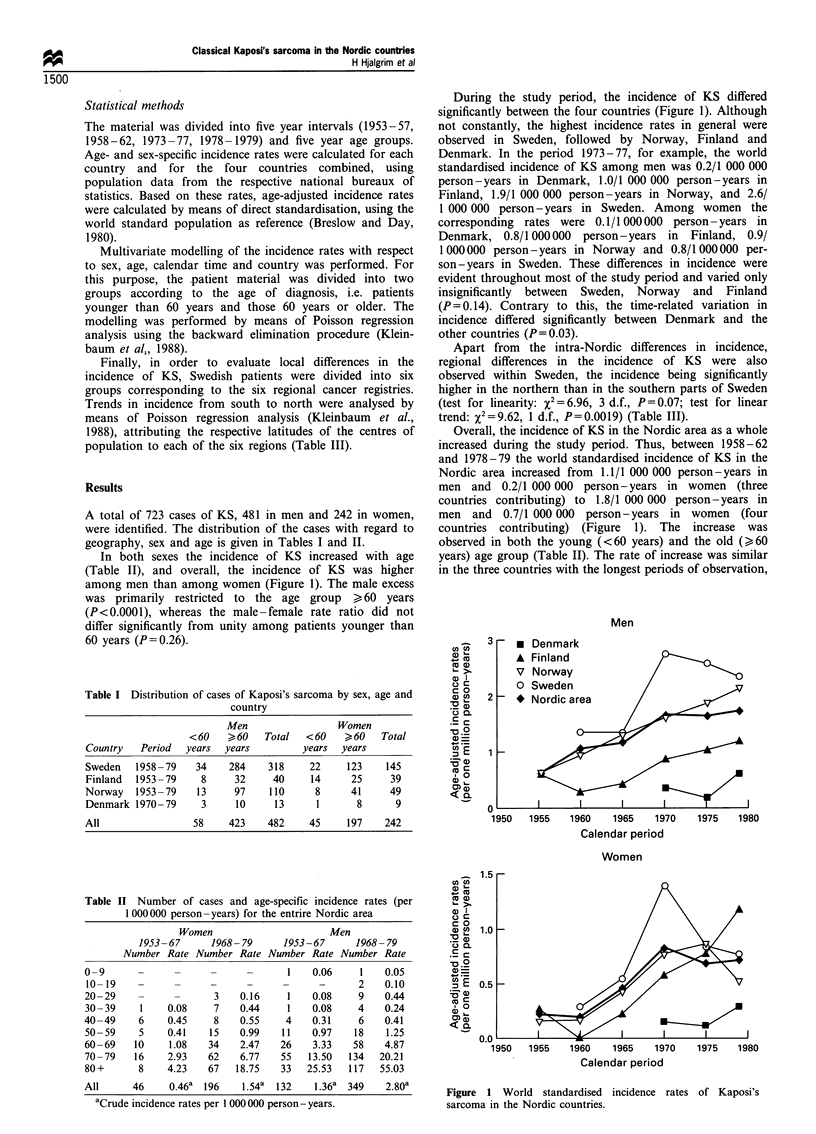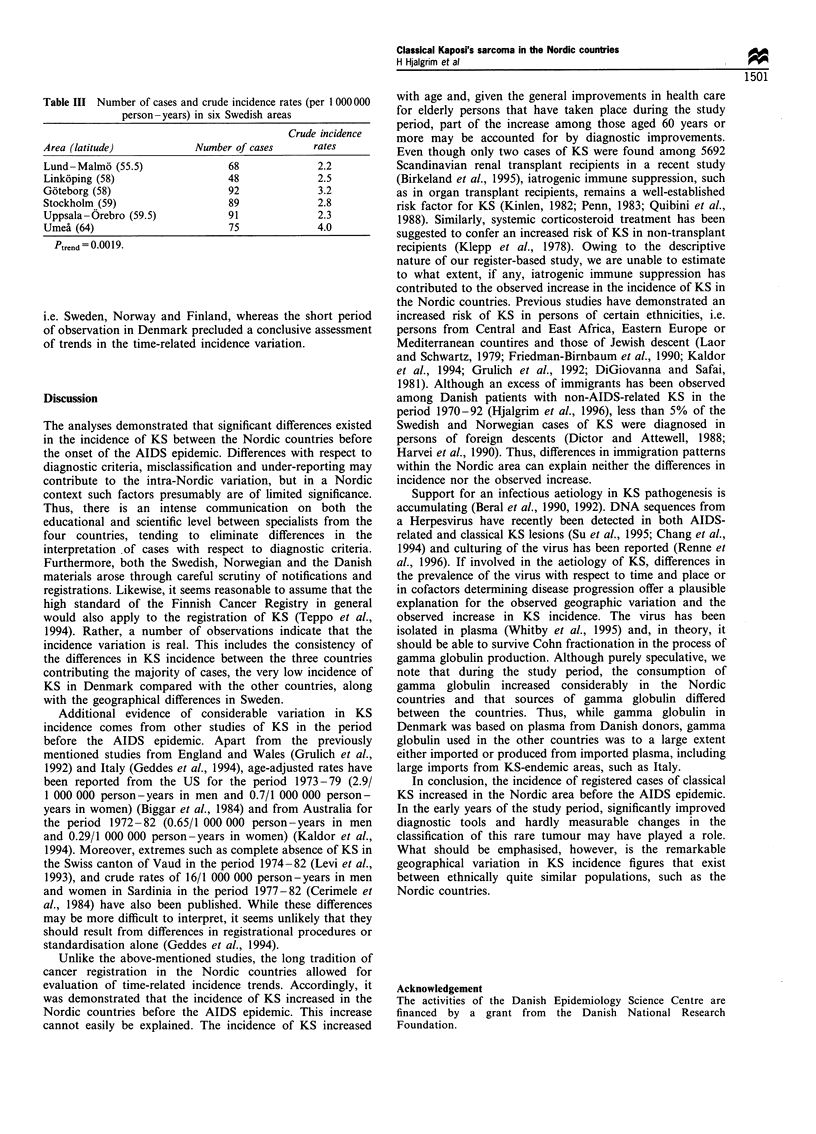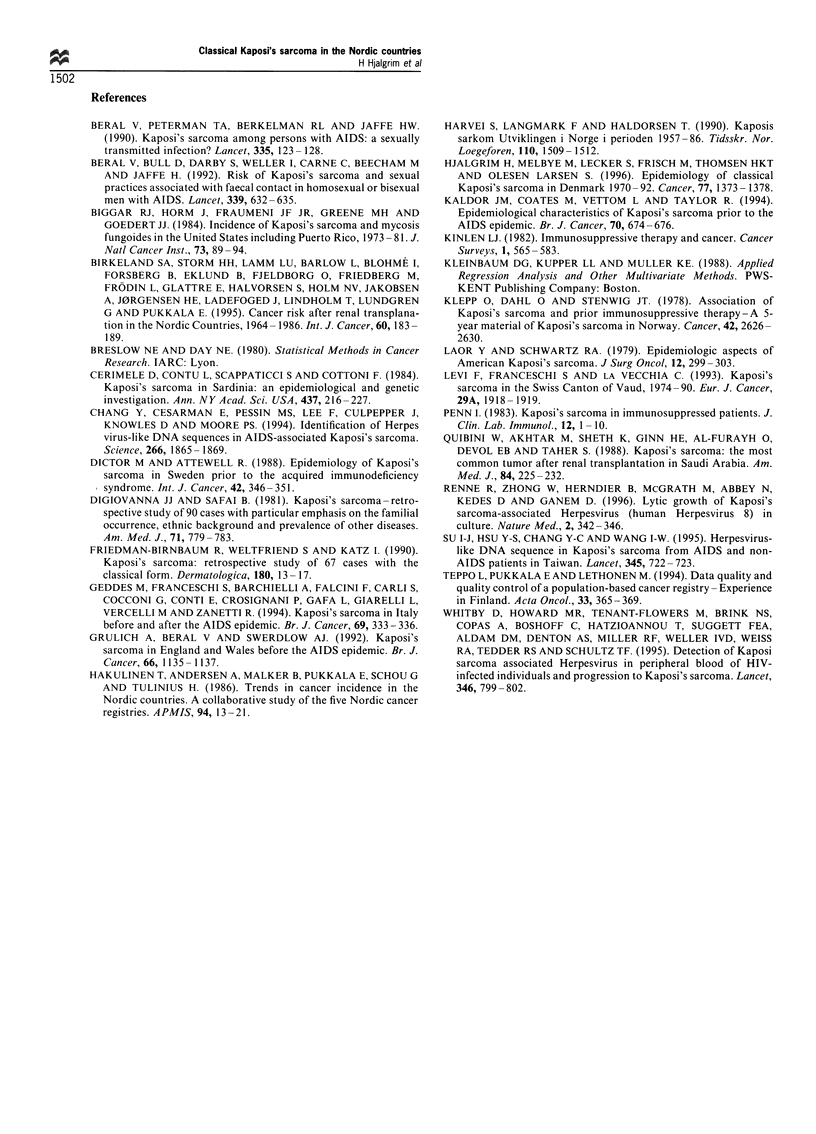# Epidemiology of Kaposi's sarcoma in the Nordic countries before the AIDS epidemic.

**DOI:** 10.1038/bjc.1996.572

**Published:** 1996-11

**Authors:** H. Hjalgrim, M. Melbye, E. Pukkala, F. Langmark, M. Frisch, M. Dictor, A. Ekbom

**Affiliations:** Danish Epidemiology Science Centre, Statens Seruminstitut, Copenhagen S, Denmark.

## Abstract

Based on data from the Nordic cancer registries, time-related trends in incidence of Kaposi's sarcoma (KS) were analysed in four ethnically similar populations before the AIDS epidemic. Data were available for different time periods in Denmark (1970-79), Sweden (1958-79), Finland (1953-79) and Norway (1953-79). KS was more common among men than among women aged 60 years or more, whereas no differences were observed for younger persons. The incidence of KS differed significantly between the four countries (P = 0.0001); Sweden having the highest and Denmark the lowest rates. Similarly, regional differences in incidence were observed within Sweden, rates being higher in the northern than in the southern areas (Ptrend = 0.002). Overall, in Nordic men the world standardised incidence rose from 0.5/1,000,000 person-years in the period 1953-57 to 1.8/1,000,000 person-years in 1978-79; in Nordic women, the corresponding rates were 0.2/1,000,000 person-years and 0.8/1,000,000 person-years respectively. The rate of increase was similar in Sweden, Finland and Norway (P = 0.14), whereas the short period of observation in Denmark precluded precise assessment of time-related incidence trends. These observations cannot be explained by registrational procedures or known risk factors for KS, and argue that environmental factors play an important role in the development of the disease.


**British Journal of Cancer (1996) 74,** 1499-1502
© 1996 Stockton Press All rights reserved 0007-0920/96 $12.00

### Epidemiology of Kaposi's sarcoma in the Nordic countries before the AIDS epidemic

H Hjalgrim[1], M Melbye[1], E Pukkala[2], F Langmark[3], M Frisch[1,4], M Dictor[5] and A Ekbom[6]

[1]*Danish Epidemiology Science Centre, Statens Seruminstitut, 5 Artillerivej, DK-2300 Copenhagen S, Denmark;* [2]*Finnish Cancer Registry, The Institute for Statistical and Epidemiological Cancer Research, 21B Liisankatu, 00170 Helsinki, Finland;* [3]*Norwegian Cancer Registry, Montebello, 0310 Oslo, Norway;* [4]*Danish Cancer Society, Division for Cancer Epidemiology, 49 Strandboulevarden, DK-2100 Copenhagen Ø, Denmark;* [5]*Department of Pathology, University Hospital, S-221 85 Lund, Sweden;* [6]*Department of Cancer Epidemiology, University Hospital, S-751 85 Uppsala, Sweden.*

**Summary** Based on data from the Nordic cancer registries, time-related trends in incidence of Kaposi's sarcoma (KS) were analysed in four ethnically similar populations before the AIDS epidemic. Data were available for different time periods in Denmark (1970–79), Sweden (1958–79), Finland (1953–79) and Norway (1953–79). KS was more common among men than among women aged 60 years or more, whereas no differences were observed for younger persons. The incidence of KS differed significantly between the four countries ($P=0.0001$); Sweden having the highest and Denmark the lowest rates. Similarly, regional differences in incidence were observed within Sweden, rates being higher in the northern than in the southern areas ($P_{trend}=0.002$). Overall, in Nordic men the world standardised incidence rose from 0.5/1 000 000 person–years in the period 1953–57 to 1.8/1 000 000 person–years in 1978–79; in Nordic women, the corresponding rates were 0.2/1 000 000 person–years and 0.8/1 000 000 person–years respectively. The rate of increase was similar in Sweden, Finland and Norway ($P=0.14$), whereas the short period of observation in Denmark precluded precise assessment of time-related incidence trends. These observations cannot be explained by registrational procedures or known risk factors for KS, and argue that environmental factors play an important role in the development of the disease.

**Keywords:** Kaposi's sarcoma; epidemiology; Nordic countries

One of the hallmarks of the onset of the AIDS epidemic in the early 1980s was the sudden increase in incidence of Kaposi's sarcoma (KS). This increase in the occurrence of a previously exceedingly rare condition naturally prompted a renewed interest in its epidemiology, including assessment of the incidence before the AIDS epidemic. From such studies, it became evident that the incidence of KS differed considerably between European countries. Thus, even taking the use of different standard populations into account, the rates reported for Italy, 10.5/1 000 000 person–years and 2.7/1 000 000 person–years in men and women respectively (Geddes *et al.*, 1994), by far exceeded the rates reported for England and Wales, being 0.14/1 000 000 person–years in both men and women (Grulich *et al.*, 1992).

Because of the rareness of KS, the quality of registry data is critically dependent on the reporting infrastructure. This, and the fact that only a few countries have longer traditions for cancer registrations, are presumably the main reasons why only a few reports have presented data for more than one decade before the AIDS epidemic. Accordingly, only a little is known about the time-related incidence trends of KS. Interestingly, however, at least one study suggested that the incidence of KS increased before the AIDS epidemic (Dictor and Attewell, 1988).

The objective of the present study was to provide further information about the epidemiology of KS in four ethnically similar Nordic populations in the pre-AIDS era. To this purpose, we took advantage of the long history and high quality of cancer registration in the Nordic countries to investigate time-related trends in classical KS and to provide incidence rates from a large north European area, populated by 22 million people in 1980.

Correspondence: H Hjalgrim
Received 26 February 1996; revised 8 May 1996; accepted 9 May 1996

### Materials and methods

The structures of the Nordic cancer registries have been described in detail elsewhere (Hakulinen *et al.*, 1986). In brief, the registries record all cases of cancers in the national populations using almost identical procedures and classifications. The ascertainment rate of the registries is high, ensuring close to 100% completeness (Hakulinen *et al.*, 1986). To avoid potential misclassification between classical KS and AIDS–KS, the present study was restricted to the period before 1980.

In Denmark, KS has been registered as a separate entity only since 1978. Cases for the period 1970–77 were sought in the Danish Cancer Registry under codes for potentially relevant skin cancers and sarcomas according to a modified version of the seventh revision of the International Classification of Diseases (ICD-7). Cases diagnosed in 1978 and 1979 were identified by searches under codes for KS and for morphologically closely related tumours, including angiosarcomas, according to the International Classification of Diseases, Oncology (ICD-O). In addition to these searches, cases were manually sought in all Danish pathology departments, applying a similar search strategy. Finally, in questionable cases the search for KS included examination of histological slides and/or scrutiny of the original pathology reports.

In the Swedish Cancer Registry, KS has been registered separately since 1958. As part of a previous study (Dictor and Attewell, 1988), all notifications of KS, reported either originally as KS or erroneously as angiosarcomas, were identified and validated.

In Norway, KS has been registered since 1953. As part of a previous report (Harvei *et al.*, 1990), cases of KS were sought under the original KS code as well as under the codes for angiosarcomas. In all cases, the histopathological descriptions were scrutinised.

The Finnish Cancer Registry has registered KS separately since the beginning of 1953. All cases of KS were sought under their original codes. Considering the high reliability of the Finnish Cancer Registry in general (Teppo *et al.*, 1994), data were accepted without further scrutiny.

*Statistical methods*

The material was divided into five year intervals (1953–57, 1958–62, 1973–77, 1978–1979) and five year age groups. Age- and sex-specific incidence rates were calculated for each country and for the four countries combined, using population data from the respective national bureaux of statistics. Based on these rates, age-adjusted incidence rates were calculated by means of direct standardisation, using the world standard population as reference (Breslow and Day, 1980).

Multivariate modelling of the incidence rates with respect to sex, age, calendar time and country was performed. For this purpose, the patient material was divided into two groups according to the age of diagnosis, i.e. patients younger than 60 years and those 60 years or older. The modelling was performed by means of Poisson regression analysis using the backward elimination procedure (Kleinbaum *et al.*, 1988).

Finally, in order to evaluate local differences in the incidence of KS, Swedish patients were divided into six groups corresponding to the six regional cancer registries. Trends in incidence from south to north were analysed by means of Poisson regression analysis (Kleinbaum *et al.*, 1988), attributing the respective latitudes of the centres of population to each of the six regions (Table III).

### Results

A total of 723 cases of KS, 481 in men and 242 in women, were identified. The distribution of the cases with regard to geography, sex and age is given in Tables I and II.

In both sexes the incidence of KS increased with age (Table II), and overall, the incidence of KS was higher among men than among women (Figure 1). The male excess was primarily restricted to the age group ⩾60 years ($P<0.0001$), whereas the male–female rate ratio did not differ significantly from unity among patients younger than 60 years ($P=0.26$).

**Table I** Distribution of cases of Kaposi's sarcoma by sex, age and country

| | | *Men* | | | *Women* | | |
|---|---|---|---|---|---|---|---|
| *Country* | *Period* | *<60 years* | *⩾60 years* | *Total* | *<60 years* | *⩾60 years* | *Total* |
| Sweden | 1958–79 | 34 | 284 | 318 | 22 | 123 | 145 |
| Finland | 1953–79 | 8 | 32 | 40 | 14 | 25 | 39 |
| Norway | 1953–79 | 13 | 97 | 110 | 8 | 41 | 49 |
| Denmark | 1970–79 | 3 | 10 | 13 | 1 | 8 | 9 |
| All | | 58 | 423 | 482 | 45 | 197 | 242 |

**Table II** Number of cases and age-specific incidence rates (per 1 000 000 person–years) for the entrire Nordic area

| | *Women* | | | | *Men* | | | |
|---|---|---|---|---|---|---|---|---|
| | *1953–67* | | *1968–79* | | *1953–67* | | *1968–79* | |
| | *Number* | *Rate* | *Number* | *Rate* | *Number* | *Rate* | *Number* | *Rate* |
| 0–9 | – | – | – | – | 1 | 0.06 | 1 | 0.05 |
| 10–19 | – | – | – | – | – | – | 2 | 0.10 |
| 20–29 | – | – | 3 | 0.16 | 1 | 0.08 | 9 | 0.44 |
| 30–39 | 1 | 0.08 | 7 | 0.44 | 1 | 0.08 | 4 | 0.24 |
| 40–49 | 6 | 0.45 | 8 | 0.55 | 4 | 0.31 | 6 | 0.41 |
| 50–59 | 5 | 0.41 | 15 | 0.99 | 11 | 0.97 | 18 | 1.25 |
| 60–69 | 10 | 1.08 | 34 | 2.47 | 26 | 3.33 | 58 | 4.87 |
| 70–79 | 16 | 2.93 | 62 | 6.77 | 55 | 13.50 | 134 | 20.21 |
| 80+ | 8 | 4.23 | 67 | 18.75 | 33 | 25.53 | 117 | 55.03 |
| All | 46 | 0.46[a] | 196 | 1.54[a] | 132 | 1.36[a] | 349 | 2.80[a] |

[a]Crude incidence rates per 1 000 000 person–years.

During the study period, the incidence of KS differed significantly between the four countries (Figure 1). Although not constantly, the highest incidence rates in general were observed in Sweden, followed by Norway, Finland and Denmark. In the period 1973–77, for example, the world standardised incidence of KS among men was 0.2/1 000 000 person–years in Denmark, 1.0/1 000 000 person–years in Finland, 1.9/1 000 000 person–years in Norway, and 2.6/1 000 000 person–years in Sweden. Among women the corresponding rates were 0.1/1 000 000 person–years in Denmark, 0.8/1 000 000 person–years in Finland, 0.9/1 000 000 person–years in Norway and 0.8/1 000 000 person–years in Sweden. These differences in incidence were evident throughout most of the study period and varied only insignificantly between Sweden, Norway and Finland ($P=0.14$). Contrary to this, the time-related variation in incidence differed significantly between Denmark and the other countries ($P=0.03$).

Apart from the intra-Nordic differences in incidence, regional differences in the incidence of KS were also observed within Sweden, the incidence being significantly higher in the northern than in the southern parts of Sweden (test for linearity: $\chi^2=6.96$, 3 d.f., $P=0.07$; test for linear trend: $\chi^2=9.62$, 1 d.f., $P=0.0019$) (Table III).

Overall, the incidence of KS in the Nordic area as a whole increased during the study period. Thus, between 1958–62 and 1978–79 the world standardised incidence of KS in the Nordic area increased from 1.1/1 000 000 person–years in men and 0.2/1 000 000 person–years in women (three countries contributing) to 1.8/1 000 000 person–years in men and 0.7/1 000 000 person–years in women (four countries contributing) (Figure 1). The increase was observed in both the young (<60 years) and the old (⩾60 years) age group (Table II). The rate of increase was similar in the three countries with the longest periods of observation,

**Figure 1** World standardised incidence rates of Kaposi's sarcoma in the Nordic countries.

**Table III** Number of cases and crude incidence rates (per 1 000 000 person-years) in six Swedish areas

| *Area (latitude)* | *Number of cases* | *Crude incidence rates* |
|---|---|---|
| Lund-Malmö (55.5) | 68 | 2.2 |
| Linköping (58) | 48 | 2.5 |
| Göteborg (58) | 92 | 3.2 |
| Stockholm (59) | 89 | 2.8 |
| Uppsala-Örebro (59.5) | 91 | 2.3 |
| Umeå (64) | 75 | 4.0 |

$P_{trend}=0.0019$.

i.e. Sweden, Norway and Finland, whereas the short period of observation in Denmark precluded a conclusive assessment of trends in the time-related incidence variation.

### Discussion

The analyses demonstrated that significant differences existed in the incidence of KS between the Nordic countries before the onset of the AIDS epidemic. Differences with respect to diagnostic criteria, misclassification and under-reporting may contribute to the intra-Nordic variation, but in a Nordic context such factors presumably are of limited significance. Thus, there is an intense communication on both the educational and scientific level between specialists from the four countries, tending to eliminate differences in the interpretation of cases with respect to diagnostic criteria. Furthermore, both the Swedish, Norwegian and the Danish materials arose through careful scrutiny of notifications and registrations. Likewise, it seems reasonable to assume that the high standard of the Finnish Cancer Registry in general would also apply to the registration of KS (Teppo *et al.*, 1994). Rather, a number of observations indicate that the incidence variation is real. This includes the consistency of the differences in KS incidence between the three countries contributing the majority of cases, the very low incidence of KS in Denmark compared with the other countries, along with the geographical differences in Sweden.

Additional evidence of considerable variation in KS incidence comes from other studies of KS in the period before the AIDS epidemic. Apart from the previously mentioned studies from England and Wales (Grulich *et al.*, 1992) and Italy (Geddes *et al.*, 1994), age-adjusted rates have been reported from the US for the period 1973-79 (2.9/1 000 000 person-years in men and 0.7/1 000 000 person-years in women) (Biggar *et al.*, 1984) and from Australia for the period 1972-82 (0.65/1 000 000 person-years in men and 0.29/1 000 000 person-years in women) (Kaldor *et al.*, 1994). Moreover, extremes such as complete absence of KS in the Swiss canton of Vaud in the period 1974-82 (Levi *et al.*, 1993), and crude rates of 16/1 000 000 person-years in men and women in Sardinia in the period 1977-82 (Cerimele *et al.*, 1984) have also been published. While these differences may be more difficult to interpret, it seems unlikely that they should result from differences in registrational procedures or standardisation alone (Geddes *et al.*, 1994).

Unlike the above-mentioned studies, the long tradition of cancer registration in the Nordic countries allowed for evaluation of time-related incidence trends. Accordingly, it was demonstrated that the incidence of KS increased in the Nordic countries before the AIDS epidemic. This increase cannot easily be explained. The incidence of KS increased with age and, given the general improvements in health care for elderly persons that have taken place during the study period, part of the increase among those aged 60 years or more may be accounted for by diagnostic improvements. Even though only two cases of KS were found among 5692 Scandinavian renal transplant recipients in a recent study (Birkeland *et al.*, 1995), iatrogenic immune suppression, such as in organ transplant recipients, remains a well-established risk factor for KS (Kinlen, 1982; Penn, 1983; Quibini *et al.*, 1988). Similarly, systemic corticosteroid treatment has been suggested to confer an increased risk of KS in non-transplant recipients (Klepp *et al.*, 1978). Owing to the descriptive nature of our register-based study, we are unable to estimate to what extent, if any, iatrogenic immune suppression has contributed to the observed increase in the incidence of KS in the Nordic countries. Previous studies have demonstrated an increased risk of KS in persons of certain ethnicities, i.e. persons from Central and East Africa, Eastern Europe or Mediterranean countires and those of Jewish descent (Laor and Schwartz, 1979; Friedman-Birnbaum *et al.*, 1990; Kaldor *et al.*, 1994; Grulich *et al.*, 1992; DiGiovanna and Safai, 1981). Although an excess of immigrants has been observed among Danish patients with non-AIDS-related KS in the period 1970-92 (Hjalgrim *et al.*, 1996), less than 5% of the Swedish and Norwegian cases of KS were diagnosed in persons of foreign descents (Dictor and Attewell, 1988; Harvei *et al.*, 1990). Thus, differences in immigration patterns within the Nordic area can explain neither the differences in incidence nor the observed increase.

Support for an infectious aetiology in KS pathogenesis is accumulating (Beral *et al.*, 1990, 1992). DNA sequences from a Herpesvirus have recently been detected in both AIDS-related and classical KS lesions (Su *et al.*, 1995; Chang *et al.*, 1994) and culturing of the virus has been reported (Renne *et al.*, 1996). If involved in the aetiology of KS, differences in the prevalence of the virus with respect to time and place or in cofactors determining disease progression offer a plausible explanation for the observed geographic variation and the observed increase in KS incidence. The virus has been isolated in plasma (Whitby *et al.*, 1995) and, in theory, it should be able to survive Cohn fractionation in the process of gamma globulin production. Although purely speculative, we note that during the study period, the consumption of gamma globulin increased considerably in the Nordic countries and that sources of gamma globulin differed between the countries. Thus, while gamma globulin in Denmark was based on plasma from Danish donors, gamma globulin used in the other countries was to a large extent either imported or produced from imported plasma, including large imports from KS-endemic areas, such as Italy.

In conclusion, the incidence of registered cases of classical KS increased in the Nordic area before the AIDS epidemic. In the early years of the study period, significantly improved diagnostic tools and hardly measurable changes in the classification of this rare tumour may have played a role. What should be emphasised, however, is the remarkable geographical variation in KS incidence figures that exist between ethnically quite similar populations, such as the Nordic countries.

**Acknowledgement**
The activities of the Danish Epidemiology Science Centre are financed by a grant from the Danish National Research Foundation.

### References

BERAL V, PETERMAN TA, BERKELMAN RL AND JAFFE HW. (1990). Kaposi's sarcoma among persons with AIDS: a sexually transmitted infection? *Lancet*, **335**, 123–128.

BERAL V, BULL D, DARBY S, WELLER I, CARNE C, BEECHAM M AND JAFFE H. (1992). Risk of Kaposi's sarcoma and sexual practices associated with faecal contact in homosexual or bisexual men with AIDS. *Lancet*, **339**, 632–635.

BIGGAR RJ, HORM J, FRAUMENI JF JR, GREENE MH AND GOEDERT JJ. (1984). Incidence of Kaposi's sarcoma and mycosis fungoides in the United States including Puerto Rico, 1973–81. *J. Natl Cancer Inst.*, **73**, 89–94.

BIRKELAND SA, STORM HH, LAMM LU, BARLOW L, BLOHMÉ I, FORSBERG B, EKLUND B, FJELDBORG O, FRIEDBERG M, FRÖDIN L, GLATTRE E, HALVORSEN S, HOLM NV, JAKOBSEN A, JØRGENSEN HE, LADEFOGED J, LINDHOLM T, LUNDGREN G AND PUKKALA E. (1995). Cancer risk after renal transplanation in the Nordic Countries, 1964–1986. *Int. J. Cancer*, **60**, 183–189.

BRESLOW NE AND DAY NE. (1980). *Statistical Methods in Cancer Research.* IARC: Lyon.

CERIMELE D, CONTU L, SCAPPATICCI S AND COTTONI F. (1984). Kaposi's sarcoma in Sardinia: an epidemiological and genetic investigation. *Ann. NY Acad. Sci. USA*, **437**, 216–227.

CHANG Y, CESARMAN E, PESSIN MS, LEE F, CULPEPPER J, KNOWLES D AND MOORE PS. (1994). Identification of Herpes virus-like DNA sequences in AIDS-associated Kaposi's sarcoma. *Science*, **266**, 1865–1869.

DICTOR M AND ATTEWELL R. (1988). Epidemiology of Kaposi's sarcoma in Sweden prior to the acquired immunodeficiency syndrome. *Int. J. Cancer*, **42**, 346–351.

DIGIOVANNA JJ AND SAFAI B. (1981). Kaposi's sarcoma–retrospective study of 90 cases with particular emphasis on the familial occurrence, ethnic background and prevalence of other diseases. *Am. Med. J.*, **71**, 779–783.

FRIEDMAN-BIRNBAUM R, WELTFRIEND S AND KATZ I. (1990). Kaposi's sarcoma: retrospective study of 67 cases with the classical form. *Dermatologica*, **180**, 13–17.

GEDDES M, FRANCESCHI S, BARCHIELLI A, FALCINI F, CARLI S, COCCONI G, CONTI E, CROSIGNANI P, GAFA L, GIARELLI L, VERCELLI M AND ZANETTI R. (1994). Kaposi's sarcoma in Italy before and after the AIDS epidemic. *Br. J. Cancer*, **69**, 333–336.

GRULICH A, BERAL V AND SWERDLOW AJ. (1992). Kaposi's sarcoma in England and Wales before the AIDS epidemic. *Br. J. Cancer*, **66**, 1135–1137.

HAKULINEN T, ANDERSEN A, MALKER B, PUKKALA E, SCHOU G AND TULINIUS H. (1986). Trends in cancer incidence in the Nordic countries. A collaborative study of the five Nordic cancer registries. *APMIS*, **94**, 13–21.

HARVEI S, LANGMARK F AND HALDORSEN T. (1990). Kaposis sarkom Utviklingen i Norge i perioden 1957–86. *Tidsskr. Nor. Loegeforen*, **110**, 1509–1512.

HJALGRIM H, MELBYE M, LECKER S, FRISCH M, THOMSEN HKT AND OLESEN LARSEN S. (1996). Epidemiology of classical Kaposi's sarcoma in Denmark 1970–92. *Cancer*, **77**, 1373–1378.

KALDOR JM, COATES M, VETTOM L AND TAYLOR R. (1994). Epidemiological characteristics of Kaposi's sarcoma prior to the AIDS epidemic. *Br. J. Cancer*, **70**, 674–676.

KINLEN LJ. (1982). Immunosuppressive therapy and cancer. *Cancer Surveys*, **1**, 565–583.

KLEINBAUM DG, KUPPER LL AND MULLER KE. (1988). *Applied Regression Analysis and Other Multivariate Methods.* PWS-KENT Publishing Company: Boston.

KLEPP O, DAHL O AND STENWIG JT. (1978). Association of Kaposi's sarcoma and prior immunosuppressive therapy–A 5-year material of Kaposi's sarcoma in Norway. *Cancer*, **42**, 2626–2630.

LAOR Y AND SCHWARTZ RA. (1979). Epidemiologic aspects of American Kaposi's sarcoma. *J Surg Oncol*, **12**, 299–303.

LEVI F, FRANCESCHI S AND LA VECCHIA C. (1993). Kaposi's sarcoma in the Swiss Canton of Vaud, 1974–90. *Eur. J. Cancer*, **29A**, 1918–1919.

PENN I. (1983). Kaposi's sarcoma in immunosuppressed patients. *J. Clin. Lab. Immunol.*, **12**, 1–10.

QUIBINI W, AKHTAR M, SHETH K, GINN HE, AL-FURAYH O, DEVOL EB AND TAHER S. (1988). Kaposi's sarcoma: the most common tumor after renal transplantation in Saudi Arabia. *Am. Med. J.*, **84**, 225–232.

RENNE R, ZHONG W, HERNDIER B, MCGRATH M, ABBEY N, KEDES D AND GANEM D. (1996). Lytic growth of Kaposi's sarcoma-associated Herpesvirus (human Herpesvirus 8) in culture. *Nature Med.*, **2**, 342–346.

SU I-J, HSU Y-S, CHANG Y-C AND WANG I-W. (1995). Herpesvirus-like DNA sequence in Kaposi's sarcoma from AIDS and non-AIDS patients in Taiwan. *Lancet*, **345**, 722–723.

TEPPO L, PUKKALA E AND LETHONEN M. (1994). Data quality and quality control of a population-based cancer registry–Experience in Finland. *Acta Oncol.*, **33**, 365–369.

WHITBY D, HOWARD MR, TENANT-FLOWERS M, BRINK NS, COPAS A, BOSHOFF C, HATZIOANNOU T, SUGGETT FEA, ALDAM DM, DENTON AS, MILLER RF, WELLER IVD, WEISS RA, TEDDER RS AND SCHULTZ TF. (1995). Detection of Kaposi sarcoma associated Herpesvirus in peripheral blood of HIV-infected individuals and progression to Kaposi's sarcoma. *Lancet*, **346**, 799–802.